# Emergence of Getah Virus Infection in Horse With Fever in China, 2018

**DOI:** 10.3389/fmicb.2019.01416

**Published:** 2019-06-20

**Authors:** Gang Lu, Jiajun Ou, Jinzhao Ji, Zixin Ren, Xue Hu, Caiying Wang, Shoujun Li

**Affiliations:** ^1^College of Veterinary Medicine, South China Agricultural University, Guangzhou, China; ^2^Guangdong Provincial Key Laboratory of Prevention and Control for Severe Clinical Animal Diseases, Guangzhou, China; ^3^Guangdong Technological Engineering Research Center for Pet, Guangzhou, China

**Keywords:** Getah virus, horses, China, phylogenetic analysis, genetic identity

## Abstract

Getah virus (GETV) is a mosquito-borne virus that was first determined in Malaysia in 1955, and can infect humans and multiple other mammals. GETV infection in horses has been reported in Japan and India, and causes great economic losses. In China, GETV has been identified in mosquitoes, pigs, foxes, and cattle with a wide geographical distribution, but has not been detected in horses. In August 2018, a sudden onset of fever was observed in racehorse in an equestrian training center in Guangdong Province in southern China. Blood samples were collected from the sick horse, and PCR/RT-PCR analysis was performed to screen for equine viral pathogens associated with fever. The results indicated that the samples were GETV RNA positive. After RT-PCR, sequencing, and assembly, the genome of the first Chinese horse-derived GETV strain, GZ201808, was obtained. Compared with the genome sequences of other GETV strains, twelve unique nucleotide substitutions were observed in GZ201808. The genome of GZ201808 had the highest genetic identity (99.6%) with AH9192, which was detected in pigs in China in 2017. Phylogenetic analysis indicated that GZ201808 clustered in Group III, and was located in an independent branch distant from other horse-derived GETV strains, indicating a unique evolutionary pattern of GZ201808. This study first determined and described the disease course of horse infected with GETV in China, sequenced and characterized the genome of the field horse-derived GETV strain, and therefore presented an unequivocal report of GETV infection in horses in China.

## Introduction

The species Getah virus (GETV) belongs to the *Alphavirus* genus of the *Togaviridae* family. Its genome is linear, positive-sense ssRNA and contains a 5′ terminal cap, two large open reading frames (ORFs), and a 3′ terminal poly(A) tract. The two ORFs encode nonstructural (nsP1 to nsP4) and structural polyproteins [C (capsid), E3, E2, 6K, E1], which are flanked by a 44 nt 26S junction region associated with transcription of an intracellular subgenomic 26S RNA (Strauss and Strauss, 1994).

As a mosquito-borne virus, GETV was first isolated from *Culex* sp. mosquitoes in Malaysia in 1955 (Griffin, 2013). Subsequent studies indicated that this virus has a broad geographical distribution, including Asia (Ksiazek et al., 1981; Morita and Igarashi, 1984; Leake et al., 1986; Li et al., 1992; Brown and Timoney, 1998; Turell et al., 2003; Bryant et al., 2005), Europe (Lvov et al., 1990), and Australia (Spradbrow, 1972), and could infect humans, monkeys, cattle, birds, pigs, horses, and other mammals (Fukunaga et al., 2000).

In China, GETV was first identified in Hainan Province in southern China in 1964, and one GETV strain designated M-1 was isolated from wild-caught mosquitoes (Li et al., 1992). Since then, several investigations have demonstrated that GETV is present in mosquitoes in China with a wide distribution, ranging between latitudes 19°N and 40°N and longitudes 97°E and 122°E (Zhai et al., 2008). In 2011, the first GETV outbreak in pigs was confirmed on a farm with piglet death in Hebei Province in northern China (Zhou et al., 2018). In addition, two GETV strains were detected in cattle and foxes in China in 2017 and 2018. The GETV E2 protein is located on the surface of viral particles, and is a glycoprotein that mediates viral entry. Evolutionary analysis of the E2 gene of GETV strains characterized worldwide since 1955 indicated that the virus has evolved into four distinct groups: Groups I–IV (Li et al., 2017). All the Chinese GETV strains were clustered in Group III with the exception of a mosquito-derived GETV strain, YN12031, detected in Yunnan Province in southern China that clustered in Group IV (Li et al., 2017).

GETV is known to be pathogenic for horses and pigs, and has resulted in large economic losses. Horses are considered an important host for the amplification and circulation of GETV (Bannai et al., 2015). To date, six GETV outbreaks in horses have been reported worldwide, five outbreaks in Japan in 1978, 1979, 1983, 2014, and 2015 (Kamada et al., 1980; Fukunaga et al., 2000; Bannai et al., 2015, 2016; Nemoto et al., 2015), and one outbreak in India in 1990 (Brown and Timoney, 1998). The clinical signs of horses with GETV infection often include pyrexia, rash on the body, and edema in the legs. Nasal discharge was only found in experimentally inoculated horses, but not in natural infection cases (Kamada et al., 1991a, b).

To the best of our knowledge, until now, there is only one example of serological evidence supporting the presence of antibodies against GETV in horses in China, with a positive rate of 4/16, which was determined in serum samples collected in Hainan Province in 1979 (Li et al., 1992). There has been no direct evidence supporting the presence of GETV in horses in China thus far. In this study, we described the disease course of racehorse infected with GETV in Guangdong Province in southern China in 2018, characterized the genetic features of the GETV detected in the sick horse and therefore presented an unequivocal report of GETV infection in horses in China.

## Materials and Methods

### Sample Collection

EDTA-treated blood samples were collected from racehorses in an equestrian training center in Guangdong Province in southern China, in which the racehorses were routinely immunized with equine influenza (Proteqflu Te, France) and Japanese encephalitis (Nisseiken, Japan) vaccines each year. After collection, the samples were immediately transported to our laboratory in an icebox for further use.

**FIGURE 1:**
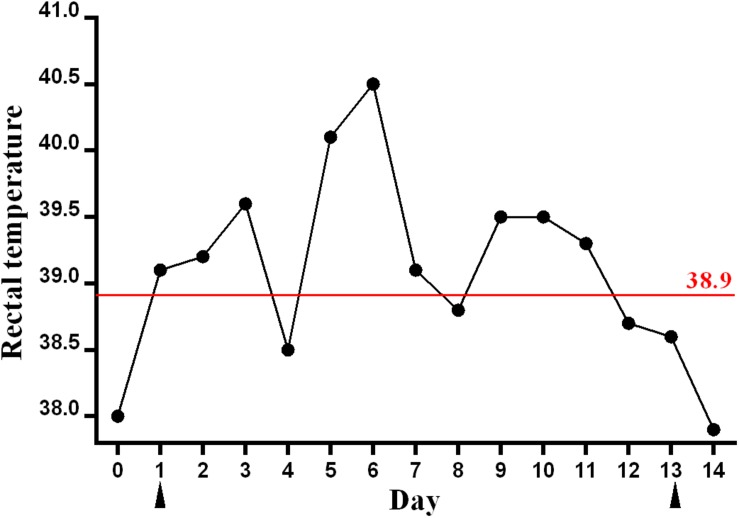
Rectal temperature of the sick racehorse infected with GETV. A rectal temperature of ≥38.9°C was considered as the reference for fever and is indicated by the red line. The blood samples were collected on days 1 and 13, which are indicated by black triangles.

In the present study, a total of 16 serum samples were collected from racehorses in the equestrian training center. Among them, two samples were collected from the sick horse on day 1 and 13, respectively. The remaining samples were collected from 14 other healthy racehorses on day 13 (Figure 1).

### Viral Detection

Total RNA/DNA of 200 μl of the samples collected from the sick horse on day 1 was extracted using a MiniBEST Viral RNA/DNA Extraction Kit (Takara, Japan) following the manufacturer’s recommendations, and finally diluted in 30 μl of nuclease-free water. A total of 6 μl of RNA was then used for reverse transcription into cDNA using a HiScript 1st Strand cDNA Synthesis Kit (Vazyme, China) with random primers. Conventional or nested PCRs targeting the conserved regions of the viral genome were then performed to detect nucleic acids of equine arteritis virus, African horse sickness virus, equine infectious anemia virus, Japanese encephalitis virus, equid herpesviruses 1 and 4, and GETV using previously published PCR primers and procedures (Table 1), and GenStar Taq Polymerase Premix (Kangrun Chengye, China). One horse serum sample tested positive for both DNA (equine parvovirus) and RNA (equine hepacivirus) viruses in our previous study was used as positive control for PCR/RT-PCR in the present study (Lu et al., 2018), and whether these two viruses were present in the serum samples collected from the sick horse was also tested (Table 1).

**TABLE 1:** Detailed information on the primers used for screening for the equine viral pathogens associated with fever.

**Virus**	**Viral**	**Targeting**	**Primer sequence (5′→3′)**	**Annealing**	**PCR**
	**genome type**	**viral gene**		**temperature**	**product**
	**(RNA/DNA)**				**length**
African horse	RNA	Segment 7	ASFV-F: GTTAAAATTCGGTTAGGATG;	55°C	1179 bp
sickness virus			ASFV-R: GTAAGTGTATTCGGTATTG		
Equine arteritis virus	RNA	ORF1b	CE^a^: TGGTAGGTGCTTCATTGGCT	42°C^a,b^	186 bp
			DE^a^: GCGGCACAAGAACACTTCTG		
			CI^b^: CCTGAGACACTGAGTCGCGT		
			DI^b^: CCTGATGCCACATGGAATGA		
Equine infectious	RNA	*gag*	P1^a^:GTAATTGGGCGCTAAGTCTAG	56°C^a,b^	211 bp
anemia virus			P2^a^: CCTCTAATAAATCTTGCTGTC		
			P4^b^:TGGGTGAATACCATACAGACA		
			P5^b^: CCAGTGGAGCATTCGGTAA		
Equid	DNA	gH	F^a^:AAGAGGAGCACGTGTTGGAT	60°C^a,b^	287 bp
herpesviruses 1			R^b^:TTGAAGGACGAATAGGACGC		
			RN^a,b^:AGTAGGTCAGGCCGATGCTT		
Equid	DNA	gB	F^a^:CTGCTGTCATTATGCAGGGA	60°C^a,b^	323 bp
herpesviruses 4			R^b^:CGTCTTCTCGAAGACGGGTA		
			RN^a,b^:CGCTAGTGTCATCATCGTCG		
Japanese	RNA	Cap	JEV19F^a^:ACTTCTTGGCTTAGTATCGT	45°C^a^;	227 bp
encephalitis virus			JEV535R^a^:GCAATGTCCGTGTTGTT	50°C^b^	
			JEV135F^b^:ATCAATATGCTGAAACGCGG		
			JEV361R^b^:CCAAGTTCTCGTTTGAAACT		
Getah virus	RNA	Cap	SagW8:CCATGGTTATTCCTGAGCTGCAAA	50°C	590 bp
			SagW9:CCACACGTCCTTTGTTGTCGAAGA		
		nsP1	M2W-S:CAGAGCATTTTCGCATCTGGCTA	50°C	434 bp
			M3W-S:ACATGAATGGAGTGGTGTCGAATCCAATCC		
Equine hepacivirus	RNA	NS3	NS3O-F^a^ ATHTGTGATGARTGCCAYAGYAC	55°C	127bp
			NS3O-R^a^ TAGTAGGTBACAGCRTTAGCYCC		
			NS3I-F^b^ TCYAARGGTGTDAAGCTTGTTGT		
			NS3I-R^b^ TGRCARAAGYTAAGRTGYCTYCC		
Equine parvovirus	DNA	VP	ak5^a^ GTCGCTGCATTCTGAGTCC	55°C	587bp
			ak^6a^ TGGGATTATACTGTCTACGGGT		
			ak7^b^ CTGCATTCTGAGTCCGTGGCC		
			ak8^b^ CTGTCTACGGGTATCCCATACGTA		

*^a^Primer/annealing temperature in the first round of nested PCR. ^b^Primer/annealing temperature in the second round of nested PCR.*

After 1.5% agarose gel electrophoresis, the PCR products with the expected size were considered positive for the corresponding viruses (Table 1). The DNA fragments were then purified using a universal DNA purification kit (Tiangen, China), cloned into the pCloneEZ vector (Clone smarter, United States), and transformed into *E. coli* DH5α competent cells (Weidi, China). After culture for 10 h, the bacterial clones were picked and detected by PCR, and the positive samples were sent for Sanger sequencing from both ends (BGI, China).

### Viral Genome Sequencing

The genome sequences of all of the GETV strains available in the NCBI database were obtained. The sequences were aligned using BioEdit 7.0.9.0^1^ with the Clustal W method, and a total of 14 primer pairs covering the near complete genome of GETV were designed using Oligo 7.0^2^ based on the conserved region among all of the GETV strains (Table 2). PCR was then performed to acquire the corresponding fragments using Phanta max superfidelity DNA polymerase (Vazyme, China). The annealing temperature for each PCR procedure was set to 55°C. After sequencing, the obtained raw data were processed using SeqMan 7.1.0 (DNAStar Lasergene package, Version 7.1.0^3^) and the genome of GZ201808 was assembled.

**TABLE 2:** Detailed information on the primers used for sequencing GZ201808.

**Primer targeting**	**Primer sequence (5′→3′)**	**Product**
**region (nt)^a^**		**length (bp)**
13–878	TGACATCACCGTTCGCTCTT;	865
	CCCTTCAGGTGAAACACGGA	
139–1017	TTTCCCGCCTTTGAGGTTGA;	879
	CATCAGGAAACCCTCTGCGT	
952–1263	AGCCCGGGCATATATGGAAAA;	312
	CGTGCCCATTTACTGAATGCT	
1166–2536	AACGCATAGTGGTGAAYGGT;	1371
	AACCACACTGCTTAGGGTCK	
2448–2723	GTGTCACTCGGGTACTTTGC;	276
	GAAGAACCGGTGGTGTCAAT	
2639-3779	TCGTGTCCACCTTGCACTAC;	1141
	TGGTCGACGCATTGTTGGTA	
3694–3889	CCTATGGATGCAGGCCGTTA;	196
	CCACCATCTCGCTGACTCTG	
3793–4948	CAGATGCTGGGAGGGGATTC;	1156
	ATGGAAAGGAGGAGCACACG	
4850–6118	CGGTACCGTGTCTATGTCGG;	1269
	TGTCGAGGCAGCTTTCAGAG	
6003–7269	GGCCGCCTGTAACTCTTTYT;	1267
	TTCATCCTGGTTGTCGTCCG	
7225–8994	TTCAAACTCGGGAAACCGCT;	1770
	CGGTAGTCAGCTGGTACGTC	
8472–9980	GAGGCCACGATGACGTGTAA;	1509
	CGTTGCGGTGTGTTCGTAAG	
9902–11091	CTGCTGCAAACCGCTWTCTT;	1190
	TGCATGTCGTTTTGRCACTG	
11059–11424	AGGTATCTGTGTGCAGTGCC;	366
	GGTTACTGGCCCYTTGAACT	

*^a^Numbered according to GETV strain AH9192 (MG865965). Y = C/T; K = G/T; W = A/T; R = A/G.*

### Viral Genome Analysis

The genome of the first GETV strain detected in horses in China, GZ201808, was aligned with 34 other GETV strains detected in mosquitoes, pigs, horses, foxes, and cattle from China, South Korea, Japan, Mongolia, and Russia (Table 3). The nucleotide and protein identity of the genome and the nonstructural and structural polyprotein coding sequences between GETV strains was calculated and displayed using MegAlign 7.1.0 (DNAStar Lasergene package, Version 7.1.0^3^; Table 4).

**TABLE 3:** Detailed information on GETV strains in China, Japan, Mongolia, South Korea, and Malaysia in this study.

**GenBank number**	**Host**	**Strain**	**Country**	**Year**
MG865965	Pig	AH9192	China	2017
KY399029	Pig	GETV-V1	China	2016
EU015062	Mosquito	HB0234	China	2002
MG865966	Pig	HNNY-1	China	2016
KY363862	Pig	HNJZ-S1	China	2011
MG865969	Pig	HNPDS-2	China	2017
MG865967	Pig	HNNY-2	China	2016
MH722255	Mosquito	JL1707	China	2017
MG865968	Pig	HNPDS-1	China	2017
MG869691	Mosquito	JL17/08	China	2017
LC152056	Mosquito	12IH26	Japan	2012
EU015063	Mosquito	YN0540	China	2005
LC079088	Horse	14-I-605-C1	Japan	2014
LC079089	Horse	14-I-605-C2	Japan	2014
KY363863	Pig	HNJZ-S2	China	2015
LC212972	Horse	15-I-752	Japan	2015
LC223131	Horse	16-I-674	Japan	2016
LC223132	Horse	16-I-676	Japan	2016
LC223130	Horse	16-I-599	Japan	2016
LC212973	Pig	15-I-1105	Japan	2015
LC107870	Mosquito	SC1210	China	2012
LC079087	Horse	MI-110-C2	Japan	1978
LC079086	Horse	MI-110-C1	Japan	1978
EF631999	Mosquito	LEIV 17741 MPR	Mongolia	2007
AY702913	Pig	South Korea	South Korea	2004
EU015061	Mosquito	M1	China	1964
AB859822	Pig	Kochi/01/2005	Japan	2005
MH722256	Cattle	JL1808	China	2018
MH106780	Fox	SD17/09	China	2017
EF631998	Mosquito	LEIV 16275 Mag	Russia	2007
MF741771	Pig	HuN1	China	2017
AB032553	Mosquito	Sagiyama virus	Japan	1956
KY434327	Mosquito	YN12031	China	2012
AF339484	Mosquito	MM2021	Malaysia	1955

**TABLE 4:** Nucleotide/amino acid identity of the genome and nonstructural and structural polyprotein coding sequences between GZ201808 and other GETV strains.

**Strain**	**Nucleotide/amino acid identity (%)**		
	**Genome**	**Nonstructural**	**Structural**
		**polyprotein**	**polyprotein**
**14-I-605-C1**	**98.8**	**98.9/99.4**	**98.6/99.3**
**MI-110-C2**	**98.5**	**98.5/99.4**	**98.4/99.4**
**15-I-752**	**98.7**	**98.8/99.3**	**98.6/99.3**
**16-I-674**	**98.7**	**98.8/99.2**	**98.5/99.2**
**16-I-676**	**98.7**	**98.8/99.2**	**98.5/99.2**
**16-I-599**	**98.7**	**98.8/99.3**	**98.5/99.2**
**MI-110-C1**	**98.5**	**98.5/99.4**	**98.4/99.3**
**14-I-605-C2**	**98.7**	**98.9/99.4**	**98.6/99.3**
M1	97.9	98.1/98.9	97.6/98.1
AH9192	99.6	99.6/99.4	99.7/99.8
GETV-V1	99.5	99.5/99.5	99.5/99.6
HB0234	98.9	99.0/99.1	98.6/99.0
HNNY-1	98.8	99.0/99.4	98.6/99.4
HNJZ-S1	98.8	98.9/99.2	98.7/99.4
HNPDS-2	98.8	99.0/99.4	98.6/99.4
HNNY-2	98.8	98.9/99.4	98.7/99.4
JL1707	98.8	98.9/99.3	98.6/99.0
HNPDS-1	98.8	99.0/99.4	98.6/99.4
JL17/08	98.8	98.9/99.4	98.6/99.2
12IH26	98.8	98.9/99.4	98.6/99.3
YN0540	98.8	98.9/99.4	98.7/99.3
HNJZ-S2	98.8	98.8/99.5	98.7/99.4
15-I-1105	98.7	98.8/99.2	98.5/99.2
SC1210	98.6	98.8/99.4	98.4/99.3
LEIV 17741 MPR	98.5	98.4/99.2	98.5/99.4
South Korea	98.3	99.1/99.5	98.9/99.3
Kochi/01/2005	97.7	97.8/99.3	97.6/99.0
JL1808	97.6	97.7/99.3	97.4/99.0
SD17/09	97.6	97.6/99.2	97.4/98.9
LEIV 16275 Mag	97.5	97.5/99.1	97.4/98.9
HuN1	97.4	97.3/99.1	97.6/98.9
Sagiyama virus	97.1	97.4/98.9	96.7/98.1
YN12031	96.2	96.2/98.6	96.3/98.4
MM2021			94.8/97.8

*The horse-derived GETV strains were indicated in bold.*

### Phylogenetic Analysis

Maximum likelihood phylogenetic trees were constructed using MEGA 7.0^4^ based on the GETV genome sequence, the nonstructural and structural polyprotein coding sequences, and the E2 gene. By “Find Best DNA Models,” the phylogenetic trees were inferred using general time reversible + gamma distributed or Tamura-Nei + gamma distributed models, based on a bootstrap value of 1,000 replicates.

### Virus Isolation

EDTA-treated blood samples collected from the sick horse on day 1 were processed for virus isolation, as described previously (Kamada et al., 1980; Bannai et al., 2015). Briefly, the plasma and leukocytes were separated, frozen and thawed three times, and then centrifuged. The supernatant was inoculated onto confluent Vero, BHK-21, RK-13, and C6/36 cell monolayers, respectively. Cultures were inspected daily for a possible change in cytopathic effects (CPE). After 5 days, both the culture and supernatant were collected, and total RNA was extracted and tested for GETV RNA by RT-PCR using two primer pairs targeting the cap and nsp1 genes. Attempts for virus isolation were also performed through intracranial inoculation of suckling mice. The mice were observed for 14 days. The brains of mice were collected and tested for GETV RNA by RT-PCR.

### Archived Serum Samples Detection

To understand whether archived horse serum samples in our laboratory contained GETV RNA, a total of 320 samples collected during 2014–2018 in our previously studies were processed for GETV detection by RT-PCR (Lu et al., 2016, 2018).

## Results

### Detecting GETV in a Racehorse With Fever

In August 2018, a sudden onset of fever was observed in a racehorse (warmblood, 17 years old) in an equestrian training center in Guangdong Province in southern China. Blood sample from the sick animal was collected after the onset of illness (day 1) (Figure 1), and tested by PCR/RT-PCR to screen for equine viral pathogens associated with fever, including equine arteritis virus, African horse sickness virus, equine infectious anemia virus, Japanese encephalitis virus, equid herpesviruses, and GETV (Table 1). This animal had been routinely vaccinated with equine influenza vaccines. No respiratory symptoms were observed in this racehorse, and equine influenza virus was not included in the screening tests.

After agarose gel electrophoresis, only two PCR products testing for the Cap and nsP1 gene of GETV showed the expected band. The subsequent sequencing and BLAST results also indicated GETV, with the highest genetic identity of 99.6% with a Chinese swine-derived GETV strain (AH9192, GenBank no. MG865965) and 99.7% with three Chinese mosquito-derived GETV strains [SC1210 (LC107810), Z12 (FJ897146), YN0540 (EU015063)], a Korean mosquito-derived GETV strain [KorL915 (JN410944)], and a Korean swine-derived GETV strain [South Korea (AY702913)] when analyzing the Cap and nsP1 genes, respectively. This GETV strain detected in a racehorse in our study was designated GZ201808, and was used for further genomic sequencing and analysis.

### Genomic Sequencing and Analysis of GZ201808

To acquire the genome of GZ201808, a gap-filling PCR strategy was used based on 14 primer pairs covering the genome of all of the published GETV strains in the NCBI database (Table 2). After sequencing and assembly, the 11,421 nt near-complete genome of the first GETV strain detected in horses in China was obtained, including a 66 nt partial 5′UTR, 7404 nt nonstructural polyprotein coding region, 44 nt 26S junction region (ATGCAGGATTACACTACATCTAAAGACCACGTATTACAG ACACC), 3762 nt structural polyprotein coding region, and 145 nt partial 3′UTR. The genome of GZ201808 has been summited in the GenBank database with accession number MK487997. Consistent with a previous description, the nonstructural polyprotein coding region of GZ201808 covers the 5′-terminal two-thirds of the viral genome (Strauss and Strauss, 1994). As determined from alignment with other GETV strains, the genome of GZ201808 encodes putative nsP1, nsP2, nsP3, nsP4, C, E3, E2, 6K, and E1 viral proteins consisting of 534, 798, 523, 611, 267, 64, 422, 61, and 438 amino acids, respectively. Among them, nsP4 is translated via readthrough of a leaky opal stop codon, UGA (Firth et al., 2011). In addition, the nucleotide length and sequence of the 26S junction region of GZ201808 were identical to those in other horse-derived GETV strains, with the exception of nucleotide substitution C→T at the last nucleotide position in some strains. The G+C content of the genome of GZ201808 (52.50%) was similar to that of other GETV strains detected in mosquitoes, horses, pigs, cattle, and foxes worldwide (52.39–52.78%). Compared with other GETV strains (Table 3), a total of 12 unique nucleotide substitutions were observed in the genome of GZ201808: G1461A, C1632T, G3291A, T3585C, G3985C, C4530T, C5037T, A5703G, C6669T, C8375T, G8972A, and A/C9494T (numbered according to GETV strain M1), causing four unique amino acid substitutions in the nsP1 (M461I), nsP2 (A746S, D769H), and C (Q75K) viral proteins, respectively. Compared with other GETV strains, the genome sequence of GZ201808 had the most nucleotide substitutions (nos.: 423) compared to YN12031, which was isolated from mosquitoes in China in 2012, and the least nucleotide substitutions (nos.: 34) compared to AH9192, which was isolated from pigs in China in 2017.

### Genetic Identity Between GZ201808 and Other GETV Strains

The nucleotide identity of the genome between GZ201808 and other GETV strains was calculated (Table 4). The results indicated that GZ201808 had a nucleotide identity of 96.2–99.6% with other GETV strains at the genome level, with the highest homology (99.6%) with AH9192 and the lowest homology (96.2%) with YN12031. The first Chinese horse-derived GETV strain, GZ201808, identified in this study had a nucleotide identity of 98.5–98.8% with other horse-derived GETV strains at the genome level, with the highest homology (98.8%) with 14-I-605-C1, which was detected in Japan in 2014, and the lowest homology (98.5%) with both GETV strains MI-110-C1 and MI-110-C2, which were detected in Japan in 1978.

When analyzing the nonstructural polyprotein coding sequence, it was found that GZ201808 had the highest nucleotide identity (99.6%) with AH9192 and the highest amino acid identity (99.5%) with three GETV strains, GETV-V1, HNJZ-S2, and South Korea, detected in pigs in China or South Korea. When analyzing the structural polyprotein coding sequence, it was observed that GZ201808 had the highest nucleotide (99.7%) and amino acid (99.8%) identity with AH9192.

### Phylogenetic Analysis of GZ201808

**FIGURE 2:**
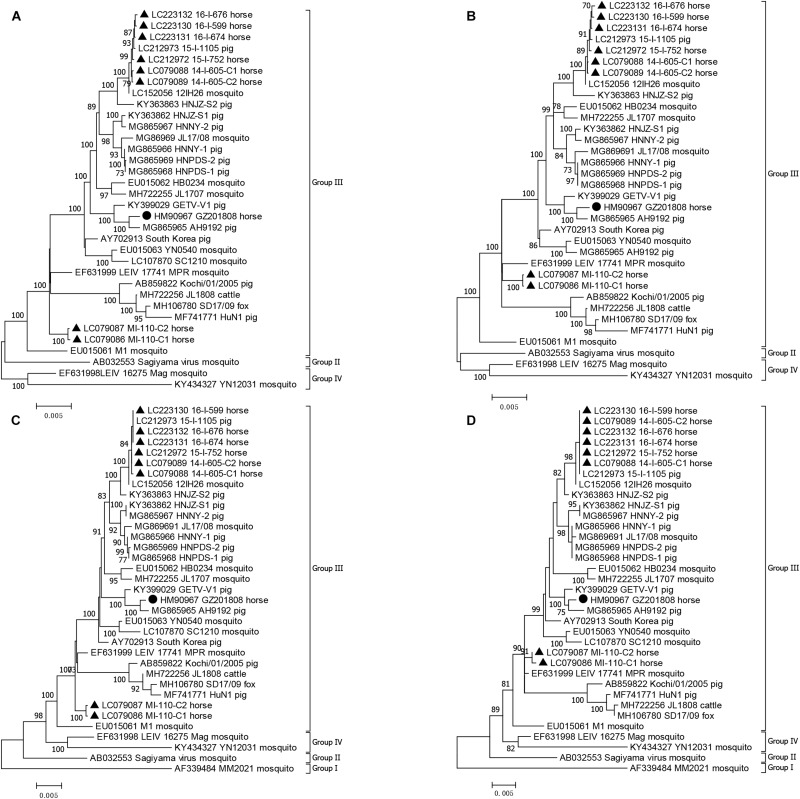
Phylogenetic analyses of GETV strains based on genome **(A)**, nonstructural **(B)** and structural **(C)** polyprotein coding sequences, and the E2 gene **(D)**. A phylogenetic tree based on the E2 gene was constructed using the TN93+G method, and three other phylogenetic trees were constructed using the GTR+G method. The GETV strain detected in this study is shown by a filled circle. The Japanese GETV strain is shown by filled triangle. The GETV strain name, country of origin, and isolation year are indicated in the phylogenetic tree.

The genome sequence, E2 gene, and the nonstructural and structural polyprotein coding sequences of GZ201808 and other GETV strains were used to construct four maximum likelihood phylogenetic trees to understand their genetic relationship (Figure 2). The phylogenetic tree based on the E2 gene had an identical topology to that identified in a previous study (Li et al., 2017). All GETV strains were rooted by the prototype GETV MM2021 in Malaysia and were classified into four groups by the E2 gene. Group III contained all of the GETV strains found worldwide after 1964, except a Chinese strain (YN12031) and a Russian strain (LEIV/16275/Mag), and included all of the GETV strains that were pathogenic to domestic animals, i.e., horses and pigs. Three other phylogenetic trees inferred from the genome sequence and the nonstructural and structural polyprotein coding sequences also indicated that GZ201808 was clustered in Group III. It was noted that Group I contained a single strain (MM2021), and only the complete structural and partial nonstructural polyprotein coding sequences of MM2021 have been published (Morita and Igarashi, 1984). In the two phylogenetic analyses based on genome and nonstructural polyprotein coding sequence, GETV strains were clustered into three groups.

In the four phylogenetic trees, it was observed that GZ201808 was located in Group III, always had the closest relationship with AH9192 and had a closer relationship with the recently determined Chinese GETV strains than with the first Chinese strain (M1).

Among the horse-derived GETV strains, it was observed that the Japanese strains detected during 2014-2016 (14-I-605-C1, 14-I-605-C2, 15-I-752, 16-I-674, 16-I-676, 16-I-599) and those in GETV outbreaks in 1978 (MI-110-C1, MI-110-C2) were located in two distant branches. The Chinese horse-derived GETV strain GZ201808 was located in an independent branch, clustered together with the pig-derived GETV strain AH9192 detected in China in 2017. In addition, the Japanese horse-derived GETV strains determined during 2014–2016 had a closer relationship with the pig-derived GETV strain 15-I-1105 detected in Japan in 2015. It indicated the horse derived GETV strains had a close relationship with the locally prevalent strains in other animals, exhibiting a distinct geographical pattern.

### Disease Course of the Infected Racehorse

To date, there has been no reported effective antiviral treatment for horses infected with GETV. During the acute and recovery stages of the disease, routine training programs were terminated, and an increase in rest time was prescribed for the sick animal.

In this study, a rectal temperature of ≥38.9°C was considered as the reference for fever in horses, which was observed in the following 10 days after day 1, with transient drops below the fever threshold applied on days 4 and 8 (Figure 1). The highest rectal temperature of this animal reached 40.5°C. The rectal temperature returned to normal after day 12. No clinical signs of nasal discharge, depression, or anorexia were observed. Blood sample was collected from the sick animal again on day 13, and was determined as GETV RNA negative by RT-PCR.

No apparent clinical sign of GETV infection was observed in other racehorses in the equestrian training center. Blood samples from the other 14 racehorses were collected on days 1 and 13, and were confirmed to be GETV negative by RT-PCR.

### Virus Isolation

After serial passages, no CPE were observed in cultured cells, and no clinical signs caused by GETV were found in suckling mice. In addition, no GETV RNA was detected in the cultured cells or mice by RT-PCR.

### Archived Serum Samples Detection

After RNA extraction, cDNA synthesis, and PCR, all the 320 archived horse serum samples were negative for GETV RNA.

## Discussion

Previously, GETV infection in horses was only recorded in Japan and India. In this study, GETV was detected for the first time in a racehorse with fever in China, which is the first description of disease caused by GETV infection in horses outside Japan and India. It has been reported that horses infected with GETV could recover spontaneously within 1 week without special treatment (Fukunaga et al., 2000), and the GETV viremia in affected horses usually lasted for only 3–5 days after onset (Kamada et al., 1991a). The short-term virus shedding period increases the difficulty of detecting GETV. In fact, before this study, considering the high detection frequency of GETV in mosquitoes and domestic animals recently in China, we analyzed 320 archived horse serum samples collected during 2014–2018 by RT-PCR (Lu et al., 2016, 2018), but found no evidence of GETV infection, although the clinical status of the tested animals was unknown. This study described GETV infection in a racehorse in China from multiple aspects, including clinical signs, pathogen confirmation, and genetic characterization, which provides a typical clinical GETV infection case in horses and is beneficial for future studies on this virus.

RNA-dependent RNA polymerase lacks proofreading/repair function and has a low fidelity (Holland et al., 1982). Accordingly, RNA viruses evolve rapidly, with a nucleotide substitution rate of close to 1 × 10^–3^ substitutions/site/year (Jenkins et al., 2002). However, a significantly lower nucleotide substitution rate was determined in vector-borne RNA viruses (Jenkins et al., 2002). The nucleotide substitution rate for the E2 gene of GETV was estimated to be 3.47 × 10^–4^ substitutions/site/year (Li et al., 2017). To sequence the genome of GZ201808, we designed 14 primer pairs based on online-published GETV genome sequences. Each primer pair matched well with the genome of GZ201808. In addition, genetic analysis between GZ201808 and other GETV strains showed a close genetic identity of >96%, which was consistent with a previous finding that GETV has been evolving slowly (Zhai et al., 2008). The reason for the low nucleotide substitution rate of GETV still needs further investigation.

A phylogenetic tree based on the E2 gene of GETV indicated that Group I rooted all GETV strains, and Group II rooted Group III and Group IV, which could be observed in the phylogenetic analysis based on the structural polyprotein coding sequence (Figure 2). Group I contained only one strain (MM2021), and its complete nonstructural polyprotein coding sequence has not been sequenced. In the phylogenetic analysis based on the nonstructural polyprotein coding sequence, Group II rooted Group IV, and Group II and Group III shared a same ancestor. In the phylogenetic analysis based on genome sequence, Group II rooted Group III, and Group III and Group IV shared the same ancestor. This analysis clearly demonstrated that three distant evolutionary patterns were involved in the genome and structural and nonstructural polyprotein coding sequence of GETV.

In this study, we attempted to isolate GETV GZ201808 *in vivo* and *in vitro* but failed. In a previously reported virus isolation experiment, a total of 209 blood samples were collected from sick horses with pyrexia in GETV outbreaks in 1978 in Japan, and only 62 virus strains were successfully isolated (Kamada et al., 1980). Of the tested cell types, Vero cells were most suitable for GETV isolation *in vitro* (Kamada et al., 1980), which were also used for virus isolation in this study. One explanation for the low successful isolation rate of GETV is strain-specific genome characteristics. For example, a total of 12 unique nucleotide substitutions were found in the genome of GZ201808, and these substitutions might influence GZ201808’s adaption and replication in Vero cells. However, the exact molecular mechanism remains to be determined. Further attempts are still needed to monitor and isolate GETV, and understand the prevalence of the virus in the horse population in China.

GETV is responsible for six major outbreaks among racehorses in Japan and India, and has caused huge economic losses. In Japan, an inactivated whole-virus vaccine (Nisseiken, Japan) was developed and used for preventing GETV infection in racehorses. In China, there is a large number of horses, and the racehorse industry is developing rapidly. To our knowledge, domestic horses in China have not been vaccinated against GETV infection. GETV is a potential threat to the racehorse industry in China. Developing a GETV vaccine using a field-prevalent strain as antigen components will help to prevent and control GETV in horses in China.

## Data Availability

All datasets generated for this study are included in the manuscript and/or the supplementary files.

## Ethics Statement

The blood sample collection method was conducted under the guidance of the South China Agricultural University Experimental Animal Welfare Ethics Committee. All procedures associated with the animal experiments were also approved by the South China Agricultural University Experimental Animal Welfare Ethics Committee.

## Author Contributions

SL: conceptualization, writing – review and editing, supervision, project administration, and funding acquisition. GL: methodology, software, formal analysis, resources, writing – original draft preparation, and visualization. JO, JJ, ZR, XH, and CW: validation. GL, JO, JJ, ZR, XH, and CW: investigation.

## Conflict of Interest Statement

The authors declare that the research was conducted in the absence of any commercial or financial relationships that could be construed as a potential conflict of interest.

## Footnotes

1 http://www.mbio.ncsu.edu/BioEdit/

2 https://www.oligo.net/

3 https://www.dnastar.com/software/genomics/

4 https://www.megasoftware.net/
