# Corrigendum: Emergence of Getah Virus Infection in Horse With Fever in China, 2018

**DOI:** 10.3389/fmicb.2019.02601

**Published:** 2019-11-14

**Authors:** Gang Lu, Jiajun Ou, Jinzhao Ji, Zixin Ren, Xue Hu, Caiying Wang, Shoujun Li

**Affiliations:** ^1^College of Veterinary Medicine, South China Agricultural University, Guangzhou, China; ^2^Guangdong Provincial Key Laboratory of Prevention and Control for Severe Clinical Animal Diseases, Guangzhou, China; ^3^Guangdong Technological Engineering Research Center for Pet, Guangzhou, China

**Keywords:** Getah virus, horses, China, phylogenetic analysis, genetic identity

In the original article, there was an error. The GenBank number of the Getah virus strain GZ201808 should be MK487997, not HM90967. In addition, the final sentence of the affected paragraph has been rewritten to make it clear that GZ201808 is the deposited genome sequence that is being determined. A correction has been made to ***Results, Genomic Sequencing and Analysis of GZ201808***.

To acquire the genome of GZ201808, a gap-filling PCR strategy was used based on 14 primer pairs covering the genome of all of the published GETV strains in the NCBI database (Table 2). After sequencing and assembly, the 11,421 nt near-complete genome of the first GETV strain detected in horses in China was obtained, including a 66 nt partial 5′ UTR, 7404 nt nonstructural polyprotein coding region, 44 nt 26S junction region (ATGCAGGATTACACTACATCTAAAGACCACGTATTACAG ACACC), 3762 nt structural polyprotein coding region, and 145 nt partial 3′UTR. The genome of GZ201808 has been summited in the GenBank database with accession number MK487997. Consistent with a previous description, the nonstructural polyprotein coding region of GZ201808 covers the 5′-terminal two-thirds of the viral genome (Strauss and Strauss, 1994). As determined from alignment with other GETV strains, the genome of GZ201808 encodes putative nsP1, nsP2, nsP3, nsP4, C, E3, E2, 6K, and E1 viral proteins consisting of 534, 798, 523, 611, 267, 64, 422, 61, and 438 amino acids, respectively. Among them, nsP4 is translated via readthrough of a leaky opal stop codon, UGA (Firth et al., 2011). In addition, the nucleotide length and sequence of the 26S junction region of GZ201808 were identical to those in other horse-derived GETV strains, with the exception of nucleotide substitution C→T at the last nucleotide position in some strains. The G+C content of the genome of GZ201808 (52.50%) was similar to that of other GETV strains detected in mosquitoes, horses, pigs, cattle, and foxes worldwide (52.39–52.78%). Compared with other GETV strains (Table 3), a total of 12 unique nucleotide substitutions were observed in the genome of GZ201808: G1461A, C1632T, G3291A, T3585C, G3985C, C4530T, C5037T, A5703G, C6669T, C8375T, G8972A, and A/C9494T (numbered according to GETV strain M1), causing four unique amino acid substitutions in the nsP1 (M461I), nsP2 (A746S, D769H), and C (Q75K) viral proteins, respectively. Compared with other GETV strains, the genome sequence of GZ201808 had the most nucleotide substitutions (nos.: 423) compared to YN12031, which was isolated from mosquitoes in China in 2012, and the least nucleotide substitutions (nos.: 34) compared to AH9192, which was isolated from pigs in China in 2017.

The authors apologize for this error and state that this does not change the scientific conclusions of the article in any way. The original article has been updated.
